# Mathematical evaluation of responses to surgical stimuli under general anesthesia

**DOI:** 10.1038/s41598-020-72307-w

**Published:** 2020-09-17

**Authors:** Shohei Ooba, Ryusuke Ueki, Nobutaka Kariya, Tsuneo Tatara, Munetaka Hirose

**Affiliations:** Department of Anesthesiology and Pain Medicine, Hyogo College of Medicine, 1-1 Mukogawa-cho, Nishinomiya, Hyogo 663-8501 Japan

**Keywords:** Physiology, Biomarkers, Signs and symptoms

## Abstract

Surgical invasion activates nociception, while anesthesia suppresses it. Under general anesthesia, stimulation, which is the balance between nociception and anti-nociception, induces responses, including activation of the autonomic nervous system. To evaluate the associations between stimulation (S) and the resultant responses (R), we examined R values, which were calculated using mathematical models of Stevens’ power law, Gompertz function and logistic function. The previously developed Nociceptive Response (NR) formula was applied as a modified logistic model. S values were calculated using a linear function in the NR formula. In a retrospective study, we developed an exponential model of Stevens’ power law and a sigmoidal model of Gompertz function using differential equations, by adjusting R values to correspond to NR values, in consecutive patients undergoing surgery under general anesthesia (n = 4,395). In a subsequent prospective study, we validated the superiority of R values of Gompertz function and the NR formula in an exponential model in adult patients undergoing tympanoplasty (n = 141) and laparoscopic cholecystectomy (n = 86). In conclusion, both modified logistic function and Gompertz function are likely appropriate mathematical models for representing responses to stimulation resulting from the balance between nociception/anti-nociception during surgical procedures under general anesthesia.

## Introduction

**Figure 1:**
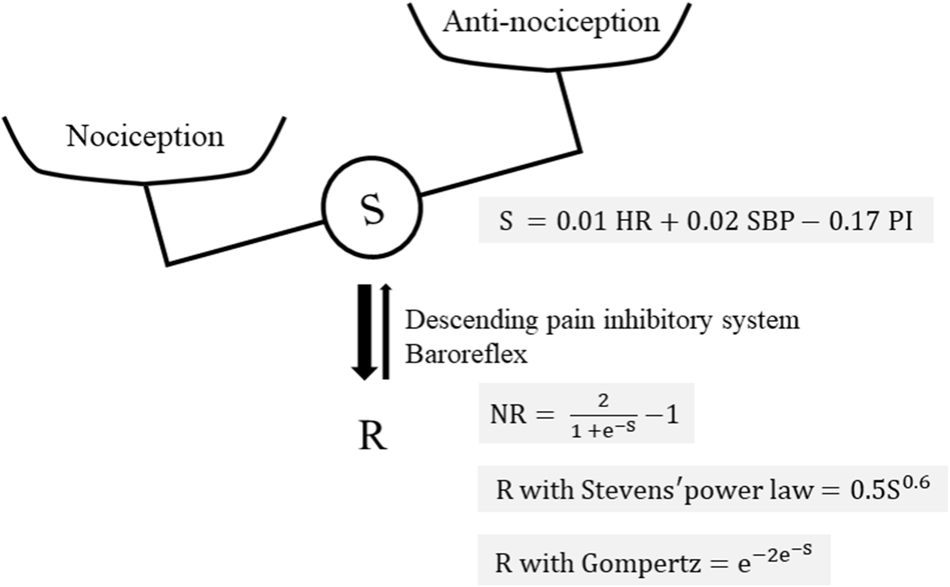
Associations between nociception/anti-nociception balance and responses. *HR* heart rate, *NR* nociceptive response, *PI* perfusion index, *R* response, *S* stimulation, *SBP* systolic blood pressure.

Nociception is the neural process of encoding and processing noxious stimuli^1^. In the conscious state, noxious stimuli cause the sensation of pain as responses. Responses to noxious stimuli during unconsciousness under general anesthesia, however, are different from those in the awake state. Surgical invasion activates peripheral nociceptors, from where nociceptive signals travel to the brain. The goal of anesthesia is to suppress nociceptive signal processing^2^. Under general anesthesia, the balance between nociception caused by surgical invasiveness and anti-nociception due to anesthetic effects determines the stimulus intensity (S), which induces physiologic responses (R), including activation of the autonomic nervous system and hypothalamic–pituitary–adrenal axis^3^. Conversely, the increases in R activate the descending pain inhibitory system, and suppress hemodynamic responses through baroreflexes^4–6^. Therefore, R values are representative of nociception and anesthetic effects, in addition to activation of several neural pathways (Fig. 1). Based on the notion that assessment of multiple physiological parameters of autonomic responses (e.g., heart rate variability, skin conductance, photoplethysmography, blood pressure, heart rate, perfusion index, etc.) would better reflect the complex nature of pain intensity and intraoperative nociception than any individual parameter alone^7^, several methods to monitor nociceptive levels have been developed^7–11^. The association between S and R calculated with these indices of nociception monitoring under general anesthesia, however, has not been well evaluated.

Stevens’ power law and the Gompertz function are mathematical models describing associations between physical stimuli and psychological responses^12,13^. These models, however, have not been applied to assess nociception levels under general anesthesia. Another mathematical model, the logistic function, was previously applied to assess responses to noxious stimuli, and the Nociceptive Response (NR) formula was developed^11^. To evaluate the association between the balance of nociception/anti-nociception and the resultant responses, we developed mathematical models using Stevens’ power law and Gompertz function, in addition to the NR formula, in a retrospective study, and validated these models in patients undergoing tympanoplasty and laparoscopic cholecystectomy in a prospective study.

## Methods

This single-institution prospective and retrospective observational study was approved by the Ethics Committee of Hyogo College of Medicine (Ethical Committee number 3138, Chairperson Koichi Noguchi) on March 4, 2019. The requirement for written informed consent for study participation was waived by the institutional ethics committee for the retrospective cohort. In the prospective cohort, informed consent was obtained using an opt-out form on our institutional web-site. This study was conducted in accordance with the principles of the Declaration of Helsinki.

### Patients

Consecutive patients of all ages, who underwent surgery under general anesthesia from May 2018 to February 2019 at our institutional surgical centre were enrolled in the retrospective study. On the other hand, adult consecutive patients aged over 20 years, American Society of Anesthesiologists-physical status (ASA-PS) class I–III, who underwent tympanoplasty or laparoscopic cholecystectomy from March 2019 to February 2020 at our institutional surgical centre were included in the prospective study. The exclusion criteria were serious preoperative comorbidities and emergency surgery in the prospective study.

### Data collection

For each patient, values of heart rate (HR), systolic blood pressure (SBP) and perfusion index (PI), that were recorded every 1 min, were obtained at 5 min before skin incision and 5, 10, 15, and 20 min after skin incision from our institutional anesthesia information management system (ORSYS, PHILIPS Japan, Tokyo, Japan). PI values were derived from the plethysmographic pulse wave amplitude via pulse oximetry (MASIMO, Irvine, CA, USA).

### Logistic function

In the logistic function, the rate of change in R to S is proportional to R, and is proportional to the ratio of R to the maximum R, which is 1 − R/R_max_ representing the suppression of R by the descending pain inhibitory system and baroreflex, and changing at the rate k^14^. Taken together, the basic differential equation for describing the logistic function is:

$$\frac{\mathrm{dR}}{\mathrm{dS}} =\mathrm{kR }\left(1-\frac{\mathrm{R}}{{\mathrm{R}}_{\mathrm{max}}}\right)$$

To solve this equation, we integrated it as follows,

$$\int \frac{\mathrm{dR}}{\mathrm{R}\left(1-\frac{\text{R}}{{\text{R}}\max}\right)}=\int \mathrm{kdS}$$

and then obtained the following logistic function,

$$\mathrm{R}=\frac{{\text{R}}\max}{{1+\left(\frac{{\text{R}}\max- \text{1} }{{\text{R}}{0}}\right)\mathrm{e}}^{-\mathrm{kS}}}$$

in which R_max_ is the maximum response, R_0_ is the minimum response, and b is a scaling factor.

Next, R was adjusted to R − 1 for maintaining nociceptive levels between 0, at a threshold of S = 0, and 1, as a maximum response. Hence the modified logistic model of NR formula, NR = R − 1, was, as obtained previously:

1 $$NR= \frac{2}{1 +{\mathrm{e}}^{-(0.01\mathrm{HR}+0.02\mathrm{SBP}-0.17\mathrm{PI})}}-1,$$

in which R_0_ = 1, R_max_ = 2, k = 1, and

2 $$S=0.01 HR+0.02 SBP-0.17 PI.$$

**Figure 2:**
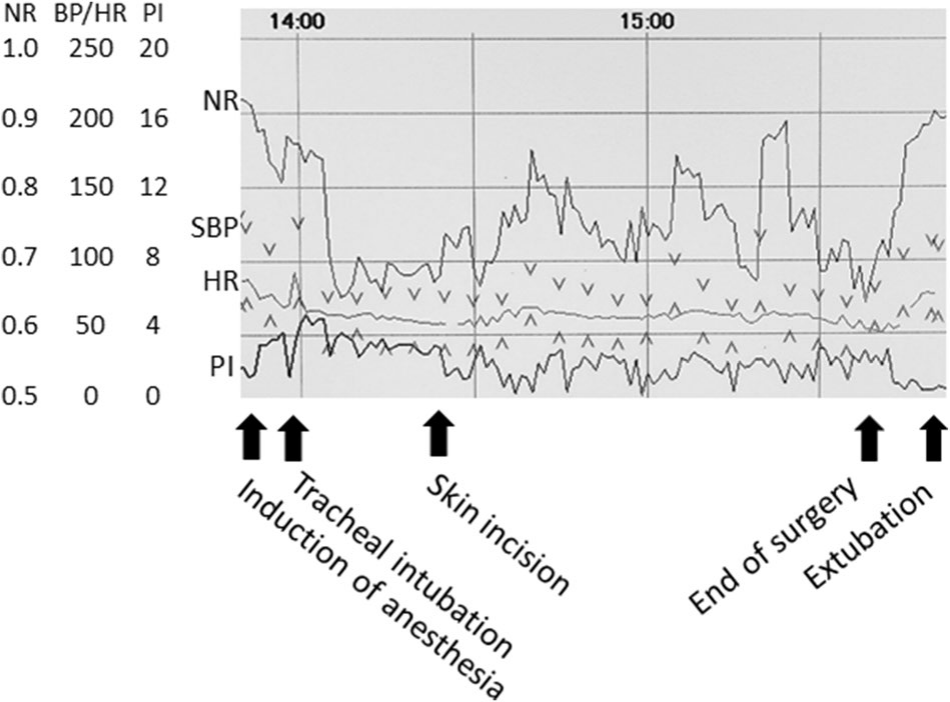
Anesthetic record showing NR, SBP, HR and PI during laparoscopic surgery. *BP* blood pressure, *HR* heart rate, *NR* nociceptive response, *PI* perfusion index, *SBP* systolic blood pressure.

Figure 2 presents an anesthetic record, showing changes in NR, HR, SBP and PI during laparoscopic surgery, where NR values were calculated every 1 min^11^.

Since higher levels of the balance between nociception/anti-nociception result in a greater increase in HR and SBP, and a lesser decrease in PI, in the previous study, the linear function of S was developed using the three variables of HR, SBP and PI to represent the balance, where intensities of surgical invasiveness during skin incision for tympanoplasty, laparoscopic cholecystectomy and open gastrectomy under general anesthesia were rated as minor, moderate and severe stimulation respectively^11^. Consequently, higher levels of the balance between nociception/anti-nociception cause higher S values.

### Stevens’ power law

In Stevens’ power law, the basic differential equation governing the change in R with respect to S in Stevens’ power law is

$$\frac{\mathrm{dR}}{\mathrm{dS}}=\mathrm{n}\frac{\mathrm{R}}{\mathrm{S}},$$

where the rate of change in R to S is inversely proportional to S and directly proportional to R^13,14^. Hence, a solution of this equation is

3 $$R=b{S}^{n},$$

in which n is an exponent that depends on the type of stimulation, and b is a scaling factor^13,14^.

### Gompertz function

The basic description of the Gompertz function is

$$\frac{\mathrm{dR}}{\mathrm{dS}}=\mathrm{AR},\quad \mathrm { where }\,\, \frac{\mathrm{dA}}{\mathrm{dS}}=-\mathrm{kA}, $$

where the rate of change in R to S is proportional to R, and the rate of change in A to S is negatively proportional to A representing the descending pain inhibitory system and baroreflex, and changing at the rate k^12,13^. The solution of this equation is

4 $$R={R}_{max}{e}^{-b{e}^{-kS}} ,$$

which is a sigmoidal function in which R_max_ is the maximum response, b is a scaling factor, and e is the Napier’s constant as Euler’s number^12,13^.

### Anesthetic managements

No premedications were prescribed before anesthesia. After general anesthesia was induced with propofol, fentanyl, and remifentanil, supraglottic airways were inserted without the use of a neuromuscular blocking drug in patients undergoing tympanoplasty. For laparoscopic cholecystectomy, rocuronium was injected intravenously to facilitate tracheal intubation. Mechanical ventilation was performed using an oxygen concentration of 40–60% to maintain normocapnea (end-tidal carbon dioxide range 35–40 mmHg). All surgeries were performed in the supine position. Anesthesia was maintained with 0.6–0.7 MAC sevoflurane or desflurane to maintain the bispectral index value between 40 and 60. Intraoperative analgesia consisted of a continuous infusion of remifentanil with additional fentanyl for the management of postoperative pain. Where needed, rocuronium was used for muscle relaxation. Regional block was not performed in any of the cases, although patients undergoing tympanoplasty received subcutaneous infiltration of 0.5% lidocaine administered by the surgeon before skin incision. All patients received standard of care treatment.

### Sample size calculation

The sample size was calculated for the prospective study using a software (PS Power and Sample Size Calculations, version 3.0, Dupont WD and Plummer WD). The calculation was done based on the assumption that a type I error probability of 0.0038 (0.05/13 ≈ 0.0038) as NR values were compared at 13 points between and within groups, and power of 0.8. From a previous study^11^, a standard deviation of NR values was 0.04, and a difference in NR between or within groups was 0.02. The ratio of the numbers of patients between two groups was estimated to be 3. Then the minimum number of patients in one group was 75.

### Statistics

Comparisons of two variables was performed using the unpaired *t*-test or chi-square test for appropriate variables, and *P* < 0.05 was considered to indicate statistical significance. One-way ANOVA followed by Tukey’s post-hoc test was used for multiple comparisons after a Bonferroni adjustment. The statistically significant level was considered as *P* < 0.0167 and *P* < 0.0033 when three tests were performed (0.05/3 ≈ 0.0167, 0.01/3 ≈ 0.0033), and *P* < 0.0038 and *P* < 0.0008 when 13 tests were performed (0.05/13 ≈ 0.0038, 0.01/13 ≈ 0.0008). Normality of data was assessed using the normal quantile plot. All statistical analyses were performed using JMS Pro version 14.2.0 (SAS Institute Inc. Cary, NC, United States). All values were reported as mean ± SD.

## Results

### Retrospective study

Table 1 shows patient characteristics in the retrospective study (n = 4,395). Both S and NR values were calculated using Eq. (2) and Eq. (1) respectively, at 5 min before skin incision and 5, 10, 15, and 20 min after skin incision. S values ranged from 0.12 to 4.73 (2.22 ± 0.52), and NR values from 0.062 to 0.983 (0.786 ± 0.95) in all patient data in the retrospective study. Since there are no absolute values of S and R during surgery under general anesthesia, we selected constant coefficients for calculation of R values in Stevens’ power law (Eq. 3) and the Gompertz function (Eq. 4) as the R values corresponding to S value changes, with almost the same values as in the NR formula, which resulted in us obtaining the following equations:

**Table 1:** Characteristics of patients in the retrospective study.

Perioperative variables	Surgery under general anesthesia n = 4,395
Age (year)	52.1 ± 24.5
Male/female (n)	2,327/2,068
Body mass index (kg·m^−2^)	22.3 ± 13.2
ASA-PS I/II/III/IV/V (n)	977/2,567/825/26/0
Elective/emergency surgery (n)	4,045/350

**Figure 3:**
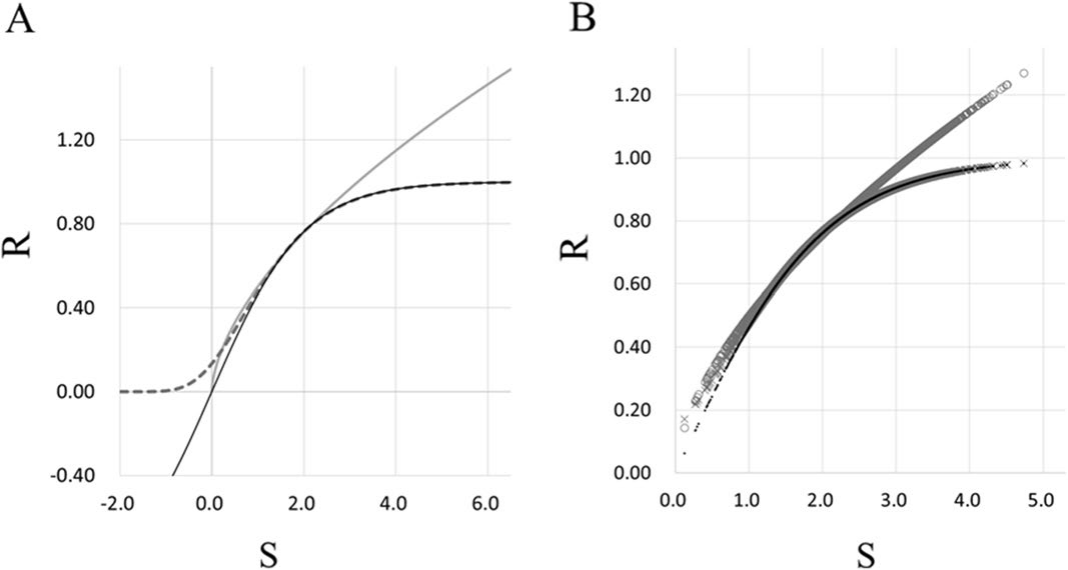
Associations between stimulation and response values in Stevens’ power law, Gompertz function, and NR formula. Three function curves of the associations between stimulation and responses with Stevens’ power law (gray line), Gompertz function (gray dotted line), and NR formula using modified logistic function (black line) are shown **(A)**. Scatter plot diagram of stimulation and responses in consecutive patients undergoing surgery under general anesthesia assessed using Stevens’ power law (gray circle), Gompertz function (gray cross), and NR formula (black dot) in retrospective study **(B)**. *NR* nociceptive response, *R* response, *S* stimulation.

*ASA-PS* American Society of Anesthesiologists-physical status.

5 $$\mathrm{R\,\, with\,\, Steven}{\mathrm{s}}^{\mathrm{^{\prime}}}\,\,\mathrm{power \,\,law}\,\,=\,\,{0.5\mathrm{S}}^{0.6},$$

where b = 0.5 and n = 0.6 in Eq. (3), and

6 $$\mathrm{R \,\,with\,\, Gompertz\,\, function}\,\,=\,\,{\mathrm{e}}^{-2{\mathrm{e}}^{-\mathrm{S}}},$$

where R_max_ = 1, b = 2, and k = 1 in Eq. (4) (Fig. 1). Figure 3A shows a function curve of values obtained by Stevens’ power law (Eq. 5), where R values increased exponentially, and also shows two function curves of the Gompertz function (Eq. 6) and the NR formula (Eq. 1), where R values increased sigmoidally. R values of Stevens’ power law and the NR formula started to increase positively at a threshold of S = 0. On the other hand, there was no threshold in the Gompertz function.

R values with Stevens’ power law ranged from 0.143 to 1.271 (0.802 ± 0.114), which were significantly higher than those with Gompertz function, which ranged from 0.171 to 0.983 (0.787 ± 0.093) (*P* < 0.001), and were also significantly higher than those with the NR formula (*P* < 0.001) (Fig. 3B). R values between Gompertz function and NR formula showed no significant difference (*P* = 0.088), and were almost identical within the clinical range of S values (Fig. 3B).

### Prospective study

Patients undergoing laparoscopic cholecystectomy (n = 86) showed significantly higher values for age and preoperative serum C-reactive protein concentrations than those undergoing tympanoplasty (n = 141) in the prospective study. Both continuous doses of remifentanil and total amounts of fentanyl used for laparoscopic cholecystectomy were also higher than those used for tympanoplasty (Table 2). Although the vasoactive agents ephedrine and phenylephrine were administered intravenously during general anesthesia in both surgeries (Table 2), these agents were not used from 5 min before skin incision to 20 min after skin incision in the prospective study.

**Table 2:** Characteristics of patient in the prospective study.

	Perioperative variables	Tympanoplasty n = 141	Laparoscopic cholecystectomy n = 86	*P* value
Preoperative variables	Age (year)	55.6 ± 16.9	60.3 ± 13.6	0.030*
	Male/female (n)	56/85	45/41	0.064
	Body mass index (kg·m^-2^)	22.5 ± 3.4	23.3 ± 4.9	0.157
	ASA-PS I / II / III (n)	48 / 89 / 4	6 / 69 / 11	< 0.001**
	Preoperative serum C-reactive protein concentration (mg·dL^−1^)	0.18 ± 0.48	0.60 ± 1.81	0.010*
Intraoperative variables	Duration of surgery (min)	121 ± 52	88 ± 35	< 0.001**
	Continuous dose of remifentanil (μg·kg^−1^·min^−1^)	0.15 ± 0.06	0.22 ± 0.07	< 0.001**
	Total amount of fentanyl (μg·kg^−1^)	2.74 ± 0.90	4.00 ± 1.72	< 0.001**
	Total amount of ephedrine (mg·kg^−1^)	10.4 ± 8.6	9.2 ± 8.0	0.196
	Total amount of phenylephrine (mg·kg^−1^)	0.14 ± 0.30	0.25 ± 0.46	0.003**

*ASA-PS* American Society of Anesthesiologists-physical status.

Significant differences at *P < 0.05, **P < 0.01.

**Figure 4:**
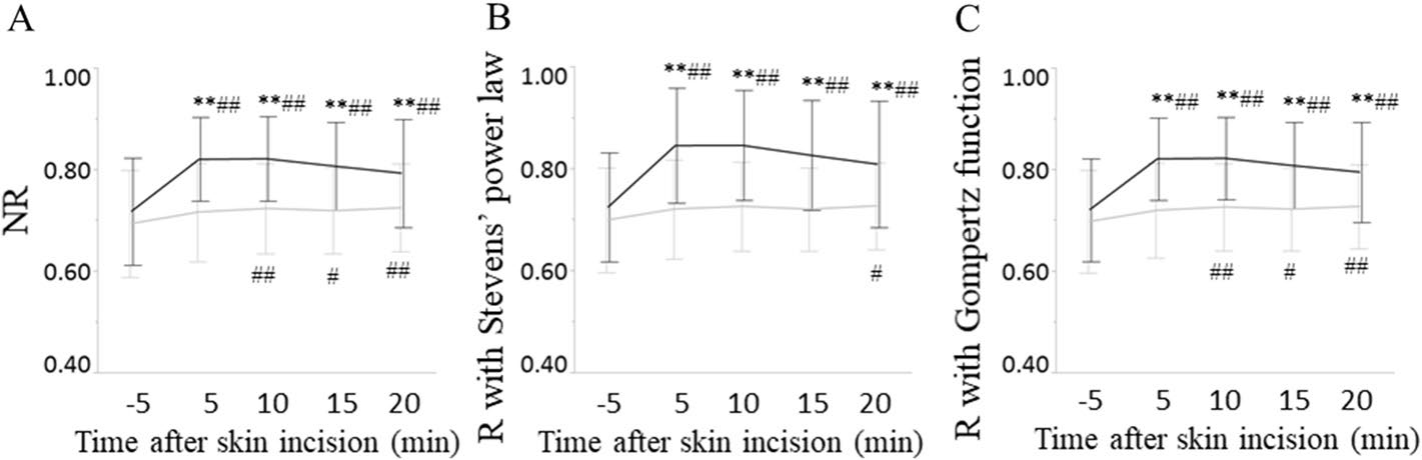
Changes in physiological responses (R) to surgical stimuli before and after skin incision. Changes in response values using the NR formula **(A)**, Stevens’ power law **(B)**, and Gompertz function **(C)** in patients undergoing tympanoplasty (gray line) and laparoscopic cholecystectomy (black line) in the prospective study. *NR* nociceptive response, *R* response. *P < 0.0038, **P < 0.0008 vs Tympanoplasty, ^#^*P* < 0.0038, ^##^*P* < 0.0008 vs before skin incision; significance using one-way ANOVA followed by Tukey’s test after Bonferroni adjustment (0.05/13 ≈ 0.0038, 0.01/13 ≈ 0.0008).

Figure 4 shows changes in R values with the three formulas before and after skin incision in the prospective study. Surgery was commenced at 0 min. In patients undergoing laparoscopic cholecystectomy, the R values of the NR formula, Stevens’ power law and Gompertz function increased significantly compared to those before skin incision (Fig. 4A–C). Furthermore, there were significant differences in R values with each mathematical model between the two patient groups. Although the R values of Stevens’ power law in patients undergoing tympanoplasty showed significant increases only at 20 min after skin incision (Fig. 4B), both R values of the NR formula and Gompertz function increased significantly at 10, 15 and 20 min after skin incision compared to those before skin incision in patients undergoing tympanoplasty (Fig. 4A, C).

## Discussion

This is the first study to mathematically evaluate responses to surgical stimulus intensity during unconsciousness under general anesthesia. In the present study, S values, which represent the balance between nociception caused by surgical invasiveness and anti-nociception due to anesthetic effects, were calculated using a linear function (Eq. 2). R values of Stevens’ power law were calculated using a power function (Eq. 5), and those of the NR formula (Eq. 1) and Gompertz function (Eq. 6) were sigmoid functions.

Surgical stimulation during skin incision for laparoscopic surgery was classified as a moderate noxious stimulus, and that for tympanoplasty was classified as a minor noxious stimulus in a previous study^11^. Given that both continuous doses of remifentanil and total amounts of fentanyl used were significantly higher in patients undergoing laparoscopic cholecystectomy than in those undergoing tympanoplasty in the present study, surgical stimulus intensity was likely higher during laparoscopic cholecystectomy than during tympanoplasty.

Although increases in R values with each mathematical model were significant after skin incision during laparoscopic cholecystectomy, those of the NR formula and Gompertz function were more significant than those of Stevens’ power law after skin incision during tympanoplasty in the present study. Even shortly after the skin incision was made, nociception caused by surgical invasion was thought to overwhelm anti-nociception due to general anesthesia during laparoscopic cholecystectomy, resulting in an immediate increase in R values. On the other hand, R values immediately after skin incision showed no changes during tympanoplasty, since subcutaneous infiltration of local anesthetics likely suppressed nociception at the time of skin incision. Surgical procedures, however, might approach the deeper tissue soon after skin incision for tympanoplasty, resulting in nociception gradually overwhelming anti-nociception. In the present study, simple exponential increases in R values with Stevens’ power law missed these small increases in R values after skin incision during tympanoplasty. Sigmoid increases in R values of the Gompertz function and the NR formula, however, were able to detect them. Previous reports stated that the physiological response to stimulation is better described as a sigmoid function, as adopted by Benjamin Gompertz than as the simple exponential function adopted by Stanley Smith Stevens^12,13^. The present study also confirmed this observation during surgery under general anesthesia.

Nociception pathways augment responses to noxious stimuli. On the other hand, both descending pain inhibitory system and baroreflex suppress them^4–6^. Therefore, these neural pathways interact each other and affect associations between S and R. Both the differential equations developed for Gompertz and logistic functions included the terms representing the suppression of R by the descending pain inhibitory system and baroreflexes, which are 1 − R/R_max_ for logistic function^14^ and dA/dS = − kA for Gompertz function^12,13^. Moreover, R values of Gompertz function were almost the same as those with the NR values using modified logistic function within the range of S values observed under the clinical conditions in the present study. Although there was a threshold of S = 0 in the NR formula, but not in the Gompertz function, the present study suggests that the sigmoid functions of these two formulas are clinically applicable for monitoring surgical stimulus intensity under general anesthesia.

Since there is no true value representing the balance between nociception caused by surgical invasion and anti-nociception induced by anesthesia, the S value of Eq. (2), which is a linear function including coefficients of HR, SBP, and PI, were thought to demonstrate the balance in the present study. The surgical pleth index (SPI) is also calculated using a linear function, the parameters of which are heart-beat interval and photoplethysmographic pulse wave amplitude^9^. Since these parameters of the autonomic nervous system change according to the descending pain inhibitory system and baroreflex^4–6^, it is inevitable that S values are inversely affected by R when evaluating changes in R values in relation to S in clinical settings under general anesthesia (Fig. 1). Given that autonomic responses are also capable of modulating peripheral nociception in addition to the descending pain inhibitory system^15^, it might be reasonable that the equations for calculation of S values include parameters of the autonomic nervous system.

Recently, artificial intelligence (AI) has been applied to anesthetic management, including monitoring of depth of anesthesia, control of anesthetic delivery, and perioperative risk prediction^16^. AI-based automated delivery of analgesics during general anesthesia, however, has not been developed so far. Since development of AI-based automated delivery of analgesics requires intraoperative nociception data as a training dataset for supervised learning, NR values and R values of Gompertz function might provide these data, which can be used for anesthesia machine development in future. Additionally, our previous study revealed that the averaged values of NR from the start to end of surgery are associated with the incidence of major complications after surgery^17^. Since higher values of mean NR during surgery correlate with higher incidence of major complications, AI-based event prediction using the training data of NR would be useful for prediction of postoperative complications.

The limitations of this study are that it was a single-institutional study and that the effects of cardiovascular agents causing changes in HR, SBP, and PI are not known. Moreover, whether other indices of nociception monitoring, including the SPI^9^, analgesia nociception index (ANI)^8^ and nociception level (NoL) index^10^, better represent S and R, are also unknown. R values during skin incision were not evaluated under severe stimulation in our prospective study. Further multi-institutional studies are needed to evaluate the effects of cardiovascular agents on R values, and to evaluate associations between the S value of Eq. (2) and the indices of SPI, ANI and NoL during surgeries with varying skin incision intensities, including all three stimulus severities, minor, moderate and severe.

In conclusion, both the NR formula and Gompertz function are likely appropriate mathematical models for representing responses to surgical stimuli under general anesthesia.

## Data Availability

The dataset analyzed during the current study are available from the corresponding author on reasonable request.
